# Species identification and cow risks of non-aureus staphylococci from South African dairy herds

**DOI:** 10.4102/ojvr.v89i1.2021

**Published:** 2022-07-27

**Authors:** Inge-Marie Petzer, Christiaan Labuschagne, Lufuno Phophi, Joanne Karzis

**Affiliations:** 1 Department of Production Animal Studies, Faculty of Veterinary Science, University of Pretoria, Pretoria, South Africa; 2 Inqaba Biotechnical Industries, Pretoria, South Africa; 3 Department of Paraclinical Science, Faculty of Veterinary Science, University of Pretoria, Pretoria, South Africa

**Keywords:** molecular epidemiology, NAS species identification, genetic diversity, cow risk factors, intramammary infection, dairy cows, South Africa

## Abstract

Detailed information on specific species of non-aureus staphylococci (NAS) has become a necessity for effective udder health control programs in South Africa. The main objective of this preliminary study was to identify the different NAS species and strains present in dairy herds in South Africa using a cost-effective method. A further objective was to investigate the effects of cow risk factors and farming systems on the NAS isolates identified. A total of 214 NAS, isolated from milk collected from 17 South African dairy herds, were identified using three diagnostic tests (API Staph test, MALDI-TOF and 16s rRNA). There was a good observed agreement between the MALDI-TOF and 16S rRNA sequencing (92.2%) and a poor observed agreement between the MALDI-TOF and API Staph (25.7%). The genetic relatedness within species was investigated in 128 of these isolates using random polymorphic amplified deoxyribonucleic acid (DNA) (RAPD), verified by multilocus sequence typing (MLST), and phylogenetic analysis and cow risk factors were investigated on species level. The main NAS species isolated were *Staphylococcus chromogenes* (75.2%), *Staphylococcus epidermidis* (9.4%) and *Staphylococcus haemolyticus* (8.9%). The RAPD test identified 34 *Staphylococcus chromogenes*, 13 *Staphylococcus epidermidis* and nine *Staphylococcus haemolyticus* strains, indicating genetic diversity amongst strains and herds. The presence of NAS intramammary infections was found to be significantly related to the farming systems, composite cow milk somatic cell count (SCC), parity and days in milk (DIM). Significantly more NAS were isolated from primiparous and from older cows. This knowledge could assist with the management of NAS on dairy farms.

## Introduction

Mastitis remains the disease that is the biggest economic challenge amongst dairy cattle in developed countries (Geary et al. 2012; Pyörälä & Taponen 2009). The subclinical form of the disease is considered the most important; it is increasing compared to clinical mastitis and is estimated to be responsible for more than 80% of economic mastitis losses (Giesecke, Du Preez & Petzer 1994; Shim, Shanks & Morin 2004), due to long-term reduction in milk quality and milk production during a cow’s lifetime. In many well-managed dairy herds, a decrease in the prevalence of intramammary infection (IMI) caused by contagious major pathogens is experienced whilst a higher proportion of subclinical or mild clinical infections caused by non-aureus staphylococci (NAS) (previously called coagulase-negative staphylococci) is present (Petzer et al. 2017). During a retrospective study, using a data set of both clinical and subclinical mastitis isolates from 830 South African commercial dairy farms, bacteria were isolated from 33.9% of 95 228 quarter milk samples examined. Of the isolates, 12.4% were major Gram-positive (6.8% *Staphylococcus aureus*, 2.1% *Streptococcus agalactiae*, 2.5% *Streptococcus uberis* and 1.0% *Streptococcus dysgalactiae*), 0.3% major Gram-negative (mainly *Escherichia coli* and *Klebsiella* spp.), 19.2% NAS and 2.0% other (Petzer et al. 2017). During the period 2018–2020, NAS were isolated from 76.5% (*n* = 59 274) of all bacteria tested at the milk laboratory of the University of Pretoria from composite cow milk samples. Thirty-five percent of the milk samples from which the NAS bacteria were isolated had a somatic cell count (SCC) in excess of 200 000 cells/mL milk, whilst 19.2% had an SCC of more than 500 000 cells/mL milk (pers. comm., Milk Laboratory, Pretoria, 2021). This data suggested that NAS is the most prevalent group of pathogens isolated from milk in South Africa, similar to other countries (Piessens et al. 2011; Sampinon et al. 2009; Taponen et al. 2006), and was the motivation for this study.

Although NAS were treated as a homogeneous bacterial group in udder health, 53 different NAS species have been identified since 2017, and 26 thereof were isolated from bovine milk (Condas et al. 2017; De Buck et al. 2021). Different NAS vary between different species in their epidemiology, pathogenicity, virulence, ecology and host adaptation, antimicrobial resistance profiles (De Buck et al. 2021; De Visscher et al. 2014; Vanderhaeghen et al. 2014, 2015) and even between different strains within the same NAS species (Piccart et al. 2016; Piessens et al. 2012). Non-aureus staphylococci have distinct relationships within the microbiome of the udder and may also have protective effects against other mastitis pathogens (De Buck et al. 2021). The main ecological niches for NAS are skin and mucous membranes (Taponen et al. 2006).

In cattle, NAS are frequently isolated from the cow’s milk, hair coat, teat skin (De Visscher et al. 2014; Piessens et al. 2011) and faeces (Wuytack et al. 2017).The most common NAS isolated from bovine milk are *S. chromogenes, S. epidermidis, S. simulans, S. haemolyticus* and *S. xylosus* (Dalen et al. 2017; Vanderhaeghen et al. 2015). Most NAS IMI, but especially *S. chromogenes,* seem to persist for 149.4 days or longer (range: 63.0–329.8 days), contrary to general beliefs (Piessens et al. 2011; Suprѐ et al. 2011). This indicates the adaption of NAS for survival in udder parenchyma.

*Staphylococci* spp. are still identified based on colony morphology, haemolysis patterns, Gram staining, catalase and coagulase production. New knowledge regarding coagulase production of NAS species has complicated such basic identification. Other methods used in species identification of NAS include the API Staph and Vitek commercial kits (BioMerieux), pulsed-field gel electrophoresis (PFGE), polymerase chain reaction (PCR), internal transcribed spacer (ITS)-PCR, MALDI-TOF mass spectrometry, malsequencing-based identification systems of the 16S rRNA, *hsp*60, *tuf, sodA*, and *rpoB* genes, multilocus sequence typing (MLST) and now whole genome sequencing (WGS) (De Buck et al. 2017; Vanderhaeghen et al. 2015). Randomly amplified polymorphic DNA (RAPD)-PCR is a simple and rapid technique used in epidemiological studies that can be performed with low quantities of DNA to identify genetic variation and establish strain-specific fingerprints (Babalola 2003; Neela et al. 2005; Olive & Bean 1999; Rabouam et al. 1999).

Non-aureus staphylococci are also classified according to their distribution, either in the cow’s environment or in cow’s milk. The size of a pan genome compared with a core genome is often an indication of adaptation of an organism to a specific niche (De Buck et al. 2017). Current knowledge suggests that *S. chromogenes* is a bovine opportunistic pathogen (Vanderhaeghen et al. 2015) and *S. epidermidis* a human-adapted species, and both species are rarely found in the environment (Piessens et al. 2011; Suprѐ et al. 2011). *Staphylococcus haemolyticus* appears to be opportunistic, as it occupies a variety of habitats (De Visscher et al. 2017; Vanderhaeghen et al. 2015). The most frequently isolated NAS from the teat skin and apex was *S. chromogenes* (Taponen, Björkroth & Pyörälä 2008; Thorberg et al. 2009). There was no known study in South Africa that identifies the specific NAS species or strains, despite the high prevalence of NAS as a potential mastitis-causing pathogen. This motivated this research. It became necessary to discover their epidemiology in order to identify the true source and risk of IMI in bovine mastitis. This preliminary study was performed to fill a gap in knowledge.

The main objective of this NAS study in South Africa was to identify the foremost NAS species up to strain level that were isolated from milk samples, using a cost-effective method which could be used in future routine diagnosis. A further objective was to investigate the effects of some cow factors and farming systems on these NAS isolates. A better understanding of NAS in South Africa and added knowledge on the effects of such cow factors on different NAS species can provide valuable information for proactive udder health management.

## Research methods and design

The study was approved by the University of Pretoria Animal Ethics Committee (reference number v046–18) as well as the Research Ethics Committee (reference number REC024–18).

## Study population

Individual cow milk samples were used from 17 dairy herds which submitted samples of all lactating cows in the herd for routine analysis. Sampling took place from April to November 2017, and the number of lactating cows per herd used in the study varied from 165 to 1493. After performing routine microbiology and cytology on the composite cow milk samples per herd, a total of 2140 NAS isolates were isolated from 11 608 samples examined. Information on the farming systems, days in milk (DIM) and parity were obtained from the records of the producers of each herd (Afimilk or Delpro management systems). Ten percent of NAS isolates, per herd, were selected at random for this study, accounting for the 214 isolates. Therefore, these NAS isolates were from cows with clinical or subclinical mastitis or with intramammary NAS infections. Parity and DIM of cows were recorded at sampling. Breeds were Holstein Friesland, Jersey and crossbreeds, and they varied between and within herds.

## Laboratory procedures

Initial bacteria isolation was performed at the South African National Accreditation System (SANAS) accredited milk laboratory (University of Pretoria, Faculty of Veterinary Science, Onderstepoort). The classical microbiological methods were performed (National Mastitis Council 2017), and initial identification of staphylococci was performed phenotypically using classic microbiology such as colony morphology, pigmentation, haemolysis, catalase, staphylase–coagulase, maltose and potassium hydroxide tests (Petzer et al. 2017). Pure colonies from these NAS isolates, after initial phenotypic identification, were frozen at −80 °C until required. Somatic cell count was performed by fluoro-opto-electronic means using a Fossomatic FC (Rhine Ruhr, Wendywood, South Africa).

### Non-aureus staphylococci species identification

Three diagnostic methods were compared for accuracy and relative cost-effectiveness for the identification of NAS species. The stored NAS isolates were revived and tested using the following methods: Matrix Assisted Laser Desorption/Ionisation Time-of-Flight Mass Spectrometry (MALDI TOF), API Staph test and 16 S rRNA sequencing.

### Matrix assisted laser desorption/ionisation time-of-flight mass spectrometry

The fresh isolates were identified using the MALDI-TOF, Bruker Daltonics, Bremen, Germany (Department of Plant and Soil Sciences, University of Pretoria, South Africa). The MALDI Biolayer 3.0 software with an integrated pattern-matching algorithm was used to compare generated peak lists against the reference library (Van Dyk et al. 2016).

### 16S rRNA test

Genomic deoxyribonucleic acid (DNA) was extracted from pure cultures using the Quick-DNA^™^ Fungal/Bacterial Miniprep Kit (Zymo Research, Catalogue No. D6005). The 16S recombinant DNA (rDNA) target region was amplified using OneTaq^®^ Quick-Load^®^ 2X Master Mix (NEB, Catalogue No. M0486) with the primers 27-F (5-AGAGTTTGATCMTGGCTCAG-3) and 1492-R (5-CGGTTACCTTGTTACGACTT-3) (Lane 1991). The PCR products were run on a gel and gel-extracted with the Zymoclean^™^ Gel DNA Recovery Kit (Zymo Research, Catalogue No. D4001). The extracted fragments were sequenced in the forward and reverse direction (Nimagen, BrilliantDye^™^ Terminator Cycle Sequencing Kit V3.1, BRD3-100/1000) and purified (Zymo Research, ZR-96 DNA Sequencing Clean-up Kit^™^, Catalogue No. D4050). The purified fragments were analysed on the ABI 3500xl and 3730xl Genetic Analyzers (Applied Biosystems, ThermoFisher Scientific). CLC Bio Main Workbench version 7.3 was used to analyse the .ab1 files generated, and species identification was obtained by a BLAST search (NCBI).

### Analytical profile index Staph test

The Analytical profile index (API) Staph test was performed according to manufacturer’s specifications and read after 24 (± 2 h). The *Staphylococcus aureus* ATCC 25923 and *Staphylococcus epidermidis* ATCC 12228 served as reference strains for quality control purposes.

### Strain typing

Random polymorphic amplified DNA analysis was performed, and preliminary phylogenetic trees were drawn on the three major groups of NAS species identified, consisting of *S. chromogenes* (*n* = 88), *S. epidermidis* (*n* = 15) and *S. haemolyticus* (*n* = 14), as the other NAS species identified from the remaining 11 isolates of the total 214 NAS isolate subset were very low in numbers (*n* = 1 or 2 each). The RAPD method was chosen due to economic constraints for this study, which consisted of a relatively large number of samples. The RAPD fingerprints were amplified using OneTaq^®^ Quick-Load^®^ 2X Master Mix (NEB,Catalogue No. M0486) with the primers B0043-17 (5-GCGATCCCCA-3), S260 (5-ACAGCCCCCA-3) and S33 (5-CAGCACCCAC-3) (Idil & Seyis Bilkay 2014) containing 0.5 µg/mL ethidium bromide (VWR Life Science, United States [US]) and visualised under UV light by Gel Doc (Cleaver Scientific, UK). The RAPD-PCR banding patterns generated were analysed manually and each informative band scored as (1) for presence or (0) for absence. Cluster analyses were carried out and dendrograms were created using the unweighted pair-group method with averaging (UPGMA) in Mega X. On the gels, a ladder was run in the first and last lane of each tier. In cases where all samples did not fit on a single gel, random samples were run again on subsequent gels to confirm patterns. Random samples were also repeated within each PCR set to verify results.

In addition, MLST was performed on a subset of samples as an added verification of the RAPD-PCR analysis. Seven *S. chromogenes* isolates (NAS-128, NAS-163, NAS-49, NAS-141, NAS-143, NAS-97, NAS-16) were selected and six housekeeping genes (*arcC, dnaJ, fumC, glpF, hutU, menF*) sequenced for each (Huebner et al. 2021). The relationships were inferred by using the maximum likelihood method (ML) and Jukes–Cantor model (Jukes & Cantor 1996) in MEGA X (Kumar et al. 2018). The percentage of trees in which the associated taxa clustered together was shown next to the branches. The tree was drawn to scale, with branch lengths measured in the number of substitutions per site. There was a total of 3637 positions in the final dataset.

## Investigation of cow risk factors

The number of NAS isolates in total and of each NAS species identified were compared between herds. This information was summarised in a table (Appendix 1, Table S3); statistical analysis could not be performed per herd due to low sample numbers.

The effects of farming systems (total mixed ration [TMR] and pasture-based systems), as well as cow factors (parity, DIM and SCC level) on the total number of NAS isolates were tested. Individually identified NAS species and strains were too few for reliable statistical testing.

## Statistical analysis

Data were captured in Excel, checked and cleaned. The one sample binomial test (procedure BNTEST) was used to test the probability of success under a binomial distribution (Freund, Mohr & Wilson 2010). The proportion calculated was the overall observed species agreement between the two tests in each case. This overall observed species agreement was carried out between the 16S rRNA sequencing method (reference method) and the MALDI-TOF test and between the 16S rRNA sequencing method and the API test.

Individually identified NAS species were too few for reliable statistical testing; thus, a one-way chi-square test was used for equal proportions per group on total number of isolates for farming systems, SCC categories, parity and DIM. If the probability level (under null hypothesis) was *p* > 0.05, then the proportions of strain categories did not differ at the 5% level (Freund et al. 2010). Data were analysed using the statistical software GenStat^®^ (VSN International 2019).

## Results

### Identification of non-aureus staphylococci

There was good overall probability of agreement of 0.973 between the 16S rRNA and the MALDI-TOF identification methods, with an exact probability, *p* < 0.001 at a 95% confidence interval (CI) for proportion (0.9313, 0.9925) (Appendix 1, Table S1). However, there was poor overall probability of agreement of 0.261 between the 16S rRNA and the API identification methods, with an exact probability *p* < 0.001 at a 95% CI for proportion (0.1906, 0.3408) (Appendix 1, Table S2). This poor observational agreement between 16S rRNA and API methods showed the same diagnosis for only 9/16, 19/86 and 3/18 for *S. epidermidis, S. chromogenes* and *S. haemolyticus*, respectively (Appendix 1, Table S2).

The most prevalent NAS species isolated from milk originating from 17 South African dairy herds using the MALDI-TOF results were *Staphylococcus chromogenes* (75.2%), followed by *S. epidermidis* (9.4%), *S. haemolyticus* (8.9%), *S. simulans* (2.8%), *S. xylosus* (2.3%) and one isolate each from *S. auricularis, S. sciuri, S. hyicus* and *S. hominis*.

*Staphylococcus chromogenes* was the most prevalent isolate in all but two herds, in which equal numbers of *S. chromogenes* and *S. epidermidis* were isolated (Appendix 1, Table S3). *Staphylococcus chromogenes* embodied 45.5% – 100.0% of NAS per herd, for the herds tested (Appendix 1, Table S3). *Staphylococcus epidermidis* and *S. haemolyticus* were both the second most prevalent NAS species, occurring in eight herds each (Appendix 1, Table S3).

**FIGURE 1:**
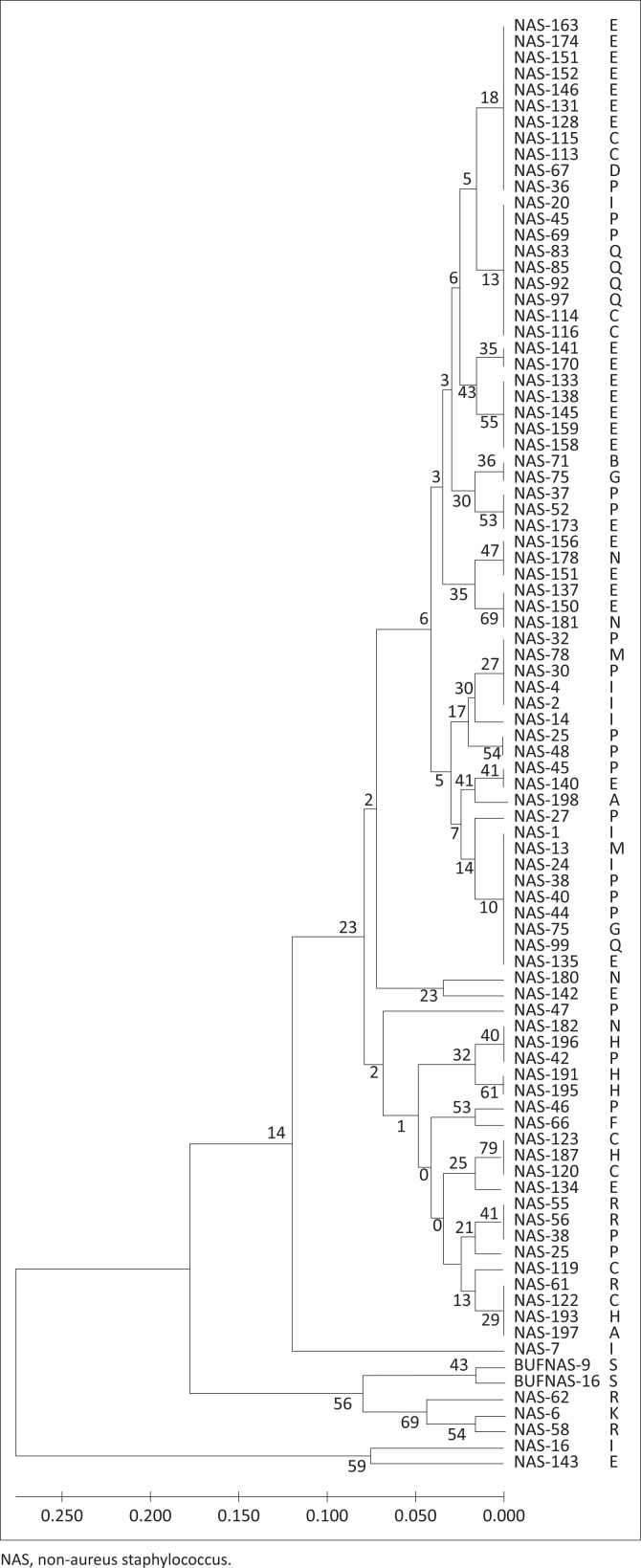
Random polymorphic amplified DNA Dendrogram of 88 *Staphylococcus chromogenes* using simple-match similarity matrix clustered by the unweighted pair-group with arithmetic mean. Letters of the alphabet indicate different herds, whilst the NAS number is the identification of the isolate tested. The dendrograms were created using the unweighted pair-group method, and the numbers on each branch indicate similarity between.

**FIGURE 2:**
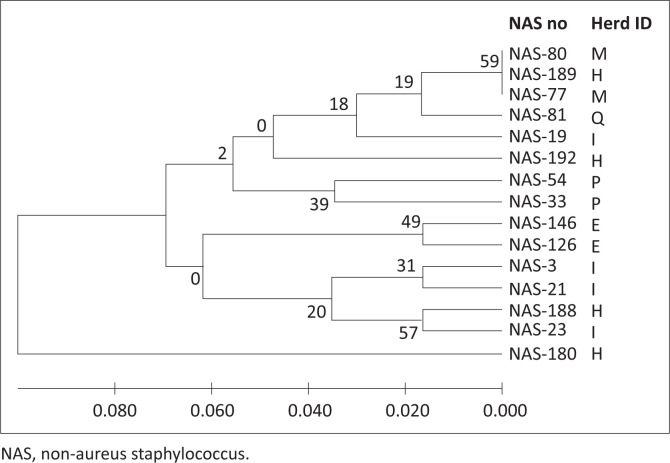
Random polymorphic amplified DNA dendrogram of 15 *Staphylococcus epidermidis*. Letters of the alphabet indicate different herds, whilst the non-aureus staphylococci sample numbers identify a non-aureus staphylococcus isolate.

**FIGURE 3:**
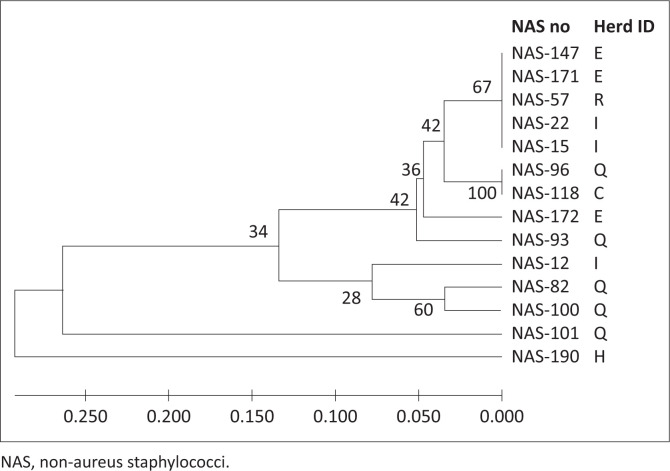
Random polymorphic amplified DNA dendrogram of 14 *Staphylococcus haemolyticus*. Letters of the alphabet indicate different herds, whilst the NAS number is the sample number.

The three foremost groups of NAS species, *S. chromogenes* (*n* = 88), *S. epidermidis* (*n* = 15) and *S. haemolyticus* (*n* = 14), were examined by RAPD-PCR with three random 10-mer primers to assess relative genetic relationships. Amplification revealed polymorphic bands ranging from 200 base pairs (bp) to 3000 bp. Isolates were scored across seven bands for primer B0043-17, nine bands for primer S33 and seven bands for primer S260. All three primer banding patterns were combined for each sample to create the dendrograms for each species (Figures 1–3). The further MLST analysis performed on a subset of seven *S. chromogenes* isolates indicated the same relationships observed for the RAPD analysis, supporting the results of the RAPD-PCR method.

### Cow risk factors

The number of NAS isolates differed per herd, for example herd L (total samples tested = 401) having only 20 isolates, from which two were selected, and herd *N* (total samples tested = 550) having 40 NAS isolates with 4 tested, of which only one NAS species was isolated (Appendix 1, Table S3).

A significant (*p* < 0.001) larger proportion of the total NAS isolates originated from pasture-based herds (70.95%) rather than from TMR based herds (29.05%) (Table 1).

**TABLE 1:** The total number and percentage of non-aureus staphylococci isolates from milk samples in pasture-based and total mixed ration farming systems.

NAS species	Number of strains	Farming system			
		Pasture	%	Total mixed ration	%
*S. chromogenes*	157	118	75.16	39	24.84
*S. epidermidis*	20	14	70.00	6	30.00
*S. haemolyticus*	19	9	47.37	10	52.63
Other NAS species	14	8	57.14	6	42.86

**Total**	**210**	**149**	**70.95**	**61**	**29.05**

Note: One-way chi-square test on total number of isolates = 36.88; degree of freedom (*df*) = 1; *p* < 0.001.

NAS, non-aureus staphylococci.

The proportions of the total NAS isolates varied significantly between level of SCC (*p* < 0.001) (Table 2). Somatic cell count levels of NAS isolates were not consistent (Table 2). All four cases of clinical mastitis identified were caused by *S. chromogenes*.

**TABLE 2:** The total number and percentage of non-aureus staphylococcus isolates from milk samples, per cow somatic cell count categories.

NAS species	Tested (*n*)	SCC							
		1–200 000 cells/mL	%	201–400 000 cells/mL	%	401–999 000 cells/mL	%	>999 000 cells/mL	%
*S. chromogenes*	157	46	29.30	17	10.83	14	8.92	80	50.96
*S. epidermidis*	20	2	10.0	2	10.0	0	-	16	80.00
*S. haemolyticus*	19	2	10.53	1	5.26	5	26.32	11	57.89
Other NAS species	14	5	35.71	0	-	0	-	9	64.29

**Total**	**210**	**55**	**26.19**	**20**	**9.52**	**19**	**9.05**	**116**	**55.24**

Note: SCC one-way chi-square test on total number of isolates = 118.42; degree of freedom (*df*) = 3; *p* < 0.001.

NAS, non-aureus staphylococcus; SCC, somatic cell count.

The proportions of the total NAS isolates per parity (lactation number) were significantly different (*p* < 0.001) (Table 3). Non-aureus staphylococci were predominantly isolated from primiparous cows (36.71%) and from third-lactation and older cows (37.68%), whilst 15.46% and 10.14% were isolated from second- and third-lactation cows respectively (Table 3).

**TABLE 3:** The total number and percentage of non-aureus staphylococcus isolates from milk samples, per parity (lactation numbers).

NAS species	Tested (*n*)	Parity							
		1st	%	2nd	%	3rd	%	> 3rd	%
*S. chromogenes*	154	61	39.61	28	18.18	13	8.44	52	33.77
*S. epidermidis*	20	3	15.00	3	15.00	3	15.00	11	55.00
*S. haemolyticus*	19	5	26.32	0	-	3	15.79	11	57.89
Other NAS species	14	7	50.00	1	7.14	2	14.29	4	28.57

**Total**	**207**	**76**	**36.71**	**32**	**15.46**	**21**	**10.14**	**78**	**37.68**

Note: Parity one-way chi-square test on total number of isolates = 50.49; degree of freedom (*df*) = 3; *p* < 0.001.

NAS, non-aureus staphylococcus.

The proportions of the total NAS isolates per DIM or stages of lactation were significantly different (*p* < 0.001) (Table 4). All NAS species tested were more frequently isolated from cows in late lactation (200 plus DIM) and the least frequently isolated from those in early lactation. On species level, 57.14% *S. chromogenes,* 60.00% *S. epidermidis,* 68.42% *S. haemolyticus*, and 71.43% other NAS species were isolated from cows in late lactation (Table 4).

**TABLE 4:** The total number and percentage of non-aureus staphylococcus isolates from milk samples per days in milk/stages of lactation.

NAS species	Tested (*n*)	Days in milk					
		1–100 days	%	101–200 days	%	> 200 days	%
*S. chromogenes*	154	27	17.53	39	25.32	88	57.14
*S. epidermidis*	20	3	15.00	5	25.00	12	60.00
*S. haemolyticus*	19	3	15.79	3	15.79	13	68.42
Other NAS species	14	2	14.29	2	14.29	10	71.43

**Total**	**207**	**35**	**16.91**	**49**	**23.67**	**123**	**59.42**

Note: DIM/stages of lactation one-way chi-square test on total number of isolates = 64.81; degree of freedom (*df*) = 2; *p* < 0.001.

DIM, days in milk; NAS, non-aureus staphylococcus.

## Discussion

Staphylococci are the most commonly isolated bacteria from milk of dairy cows (Condas et al. 2017). When performing routine mastitis diagnosis, staphylococci are usually divided into NAS and coagulase-positive staphylococci (*S. aureus*) (Taponen & Pyörälä 2009). According to Zadoks and Watts (2009), the accurate identification of NAS at species level cannot be provided reliably by classical bacteriology using phenotypic and biochemical criteria alone.

## Prevalence and species identification of non-aureus staphylococci

A broad variation in colony morphology and colour were observed in 214 NAS isolates originating from 17 South African commercial dairy herds, decreasing the discriminative power of this method of identification and species typing. The MALDI-TOF method was selected as the preferred method for routine diagnosis of NAS to species level in South Africa, as its results compared favourably with those of the 16S rRNA sequencing method (reference method used), and it was the most cost-effective test in South Africa at the time. There was good overall probability of agreement of 0.973 between the 16S rRNA and the MALDI-TOF identification methods, with an exact probability *p <* 0.001 at a 95% CI for proportion (0.9313, 0.9925). This can be seen by the diagonal pattern highlighted in the table of agreement (Table 2), showing the same diagnosis for 16/17, 99/100 and 16/17 for *S. epidermidis, S. chromogenes* and *S. haemolyticusi*, respectively. Although very few numbers were isolated of *S. hyicus, S. sciuri, S. simulans* and *S. xylosus*, this table showed that both the 16S rRNA and MALDI-TOF method picked up these species (Table 2).

However, there was poor overall probability of agreement of 0.261 between the 16S rRNA and the API identification methods, with an exact probability *p <* 0.001 at a 95% CI for proportion (0.1906, 0.3408) (Appendix 1, Table S1). This finding agreed with other studies which showed that phenotypic tests (API Staph ID 32 and Staph-Zym) were inaccurate in species identification of NAS from bovine milk samples, and genotypic methods were shown to have higher typing ability and accuracy in the identification of bovine NAS (De Buck et al. 2021; Sampimon et al. 2009; Taponen & Pyörälä 2009). API Staph has also been shown to have a moderate to low performance in goat NAS identification (Koop et al. 2012).

The overall predominant NAS species in this study was *S. chromogenes*, followed by *S. epidermidis* and *S. haemolyticus. Staphylococcus simulans* and *S. xylosus* were isolated at lower frequencies and *S. hominis, S. hyicus* and *S. sciuri* in < 0.5% (Appendix 1, Table S3).

The results of this study relate to the findings of Piessens et al. (2011) that identified *S. chromogenes* as the most prominent NAS and *S. epidermidis, S haemolyticus* and *S. simulans* as frequently isolated by other studies (Dalen et al. 2017; Vanderhaeghen et al. 2015). The three species of NAS isolates identified in this study agreed with the findings from other studies conducted in countries such as Canada, Belgium (4 studies), Finland, the Netherlands, the United States, Poland and Argentina (Condas et al. 2017; De Visscher et al. 2016, 2017; Jenkins et al. 2019; Valckenier et al. 2021). The prevalence of each species of NAS in this study (Appendix 1, Table S3) differed from that found in a study performed in Switzerland, which found that the most prevalent NAS species were *S. xylosus* (35%), *S. vitulinus* (10%) and *S. chromogenes* (7%), whereas *S. chromogenes* (5%), *S. xylosus* (5%) and *S. haemolyticus* (4%) isolates were least prevalent (Jenkins et al. 2019). Less prevalent NAS species such as *S. simulans* and *S. xylosus* were isolated in higher frequencies by Suprѐ et al. (2011). *Staphylococcus chromogenes* was the most predominant species isolated from the 17 South African herds and may complicate basic microbiology diagnostics, as its coagulase results are inconstant (Wuytack et al. 2017).

Similar to this study, other studies also used 16S rRNA sequencing to identify NAS species successfully (Casaes Nunes et al. 2016).

As shown in Figures 1–3 of this study, previous similar studies also determined evolutionary relationships of NAS strains by the construction of dendrograms (Travesari et al. 2019). The different NAS species and strains in this study indicated that these herds contained genetically diverse strains, which agreed with the results of two other studies which also showed genetic diversity within strains and between herds (Koop et al. 2012; Naushad et al. 2019).

## Strain typing

Great diversity in genotypes of the 88 *S. chromogenes* isolates was indicated, with 34 different strains in total and 17 groups of closely related strains (Figure 1). Group one consisted of 13 identical strains and a further strain which was different but closely related to group one (Figure 1). The largest cluster of 11 isolates originated from four different herds. The maximum likelihood phylogeny, drawn from sequencing data for the subset of 7 *S. chromogenes* isolates on which MLST analysis was performed, reflected the same relationships observed from the RAPD-PCR analysis. This MLST analysis was performed in order to validate the RAPD dendrograms which otherwise may include possible bias, as explained in the study by Van Belkum et al. (2001), which compared the accuracy of strain typing between various genetic methods. In both analyses, isolates NAS-128 and NAS-163 as well as NAS-48 and NAS-97 fell together as the same strain, whilst NAS-141 fell closer to these two clusters than NAS-16 and NAS-143, which fell on a separate branch and were more dissimilar compared to the other isolates. This showed similar genetic diversity to the study by Jenkins et al. (2019), in which 26 different strains were found out of a total of 48 isolates for *S. chromogenes*.

Genetic diversity amongst the 15 *S. epidermidis* isolates was evident as 13 potentially different strains (Figure 2) were present. The *S. epidermidis* formed two clusters of similar and one dissimilar isolate (Figure 2). These findings were also similar to those of Jenkins et al. (2019), who found that amongst the 13 *S. epidermidis* isolates tested, each of them was a different strain. The 14 *S. haemolyticus* isolates consisted of nine different strains, indicated by two closely related clusters as well as two dissimilar isolates (Figure 3). These results were also similar to the results obtained by Jenkins et al. (2019) for *S. haemolyticus*, which showed wide genetic diversity with the strains clustered into six major groups and multiple subgroups.

The RAPD-PCR analysis indicated that several strains of *S. chromogenes, S. epidermidis* and *S. haemolyticus* were isolated, with similar strains originating from different herds as well as different strains being isolated from the same herd (Figures 1–3). The different NAS species and strains isolated from South African dairy herds showed broad genetic diversity, which agreed with the results of a study in the United States of America (Jenkins et al. 2019) and with a review study showing similar worldwide results (De Buck et al. 2021).

## Interherd relationship of non-aureus staphylococci species

The number of isolates per herd, as indicated in Appendix 1, Table S3, were 10% of the total number of NAS isolated per herd. The number of NAS species per herd was too low for statistical analysis on the interherd relationship (Appendix 1, Table S3). However, the table indicated different NAS species per herd, and *S. chromogenes* was the most prominent in all herds (Appendix 1, Table S3). This agreed with the study by Travesari et al. (2019), which also found differences in NAS species distribution between herds. Similar to other studies (De Buck et al. 2021; Idil & Seyis Bilkay 2014; Jukes & Cantor 1996; Naushad et al. 2019; Piessens et al. 2011), the results of this study showed wide diversity not only between NAS species but also between different NAS strains within the same species for *S. chromogenes, S. epidermidis* and *S. haemolyticus*, both amongst different herds and within the same herd (Appendix 1, Table S3 and Figures 1–3). This suggested a potentially large natural reservoir of NAS species and strains, suggesting that cow risk factors on a herd level may be involved in the establishment of particular species in a dairy herd (De Buck et al. 2017; Piessens et al. 2011). The same authors (Piessens et al. 2011) showed that the primary reservoirs of the NAS species that cause IMI varied. *Staphylococcus chromogenes* and *S. epidermidis* were rarely found in the environment, indicating that other reservoirs were more important in their epidemiology. However, for *S. haemolyticus* and *S. simulans*, the environment was found as a reservoir, suggesting that IMI with these species were possibly environmental in origin (Piessens et al. 2011).

## Farming systems

A significantly larger proportion (*p* < 0.001) of the total NAS isolates originated from pasture-based herds (71.0%) than from TMR-based herds (29%) (Table 1). Proportionally more *S. chromogenes* was isolated from pasture-based (75.16%) than TMR herds (24.84%), whilst *S. haemolyticus* was more prevalent among TMR herds (Table 1). Strains of *S. chromogenes* seemed to vary both within and between herds and farming systems (Appendix 1, Table S3 and Figure 1). These findings were in general agreement with those of Taponen et al. (2016). A study by Jenkins et al. (2019) showed a variation in geographical distribution of the different NAS species, and the three major species that occurred independent of geographical region with the highest frequencies were *S. chromogenes, S. haemolyticus* and *S. simulans*.

## Somatic cell counts

The proportions of the total NAS isolates per SCC group were significantly different (*p* < 0.001) (Table 2). This finding agreed with a comparison observed between the average SCC of milk samples from which specific NAS species were isolated and the number of exo-enzyme, host evasion and iron uptake genes these species carried (Condas et al. 2017; Naushad et al. 2019). In addition, these studies showed interesting associations between virulence genes identified in NAS, with marked differences in the strength of these associations between isolates with low SCC and clinical mastitis NAS isolates (De Buck et al. 2021 Naushad et al. 2019). However, the SCC of different NAS species were not consistent, which could have been attributed to many of the other factors affecting SCC. Thus, the different NAS species and strains did not show significant trends in SCC (Table 2).

There is increased pressure to achieve lower bulk tank SCC levels and in turn benefit from the associated milk quality premiums (Sears & McCarthy 2003). Although most subclinical NAS IMI are not treated, they are the most prevalent bacteria isolated from milk samples in South Africa (Petzer et al. 2017) and other countries (De Buck et al. 2017; Monteiro et al. 2017). The amount of NAS isolates identified in South Africa has increased from 6.2% to 38.0% of milk samples when all lactating cows in a herd are cultured (Petzer et al. 2017). The SCC level of the NAS positive composite cow milk samples varied considerably, from 2000 cells/mL milk to exceeding one million cells/mL milk. Herds with 35% NAS IMI may have had SCC distribution of more than 91% of cows with SCC below 250 000 cell/mL milk, whilst in other herds with similar bulk tank SCC levels, there was 10.0% NAS IMI and 45.0% of cows had SCC at levels below 250 000 cells/mL milk. Non-aureus staphylococci are known to increase the SCC threefold or fourfold and decrease the quality of milk (Leitner & Blum 2017; Taponen & Pyörälä 2009). Bacterial cure rate for NAS IMI treated with antimicrobials was significantly higher (86.0%) than in non-treated quarters (46.0%). However, antimicrobial resistance was more common for NAS than for *S. aureus* (Taponen et al. 2006). There was a need for a cost-effective method of identification of NAS species that could form part of the current routine diagnostic portion of a proactive udder health program to investigate one of the reasons why there was such a large variation in SCC with NAS IMI.

## Parity

The proportions of the total NAS isolates per lactation number (parity) were significantly different (*p < 0.001*) (Table 3). The findings that NAS were isolated more frequently from first lactation cows (36.71%) and older cows (≥ 3 lactations) (37.68%) were different from other studies. Sampimon et al. (2009) and Tenhagen et al. (2009) found NAS to be more common in primiparous than in multiparous cows. *Staphylococcus chromogenes* was more prominent in primiparous cows and in cows in their second lactation, whilst similar to the findings of Sampimon et al. (2009), *S. epidermidis* and *S. haemolyticus* were more frequently isolated from the older cows (≥ 3 lactations).

## Days in milk

The proportions of the total NAS isolates per lactation days group or DIM were significantly different (*p* < 0.001) (Table 4). All NAS species in this study were more frequently isolated from cows in late lactation (200 days plus) and the least from early lactation cows (Table 4). These findings were in contrast to those of Jenkins et al. (2019), which found that fewer numbers of *S. chromogenes, S. haemolyticu*s and *S. epidermidis* were isolated from cows in late lactations compared to early lactations, although these were not significantly different.

## Conclusion

This preliminary study for South Africa identified NAS to species level using three different methods. There was a good observed agreement between species identification of MALDI-TOF and 16S rRNA sequencing and a poor observed agreement with the phenotypic API Staph test. The MALDI-TOF was found to be the most cost-effective method to be used in future for routine NAS species identification under South African conditions. The overall predominant NAS species isolated in high frequencies from South African dairy herds were *S. chromogenes, S. epidermidis* and *S. haemolyticus.* The RAPD-PCR analysis, validated by MLST for strain level identification of NAS, showed wide genetic diversity of various strains of *S. chromogenes, S. epidermidis* and *S. haemolyticus* isolated between different herds and within the same herd. No clear patterns of origin of particular strains could be deduced, which highlighted the extensive genetic diversity of the NAS isolated from herds in this study.

There was a significantly larger proportion of the total NAS isolates originating from pasture-based than from TMR herds. The proportions of the total NAS isolates varied significantly between the level of SCC, parity and DIM. Knowledge of the effect of these cow factors on NAS can assist in the improvement of udder health management of dairy herds affected by NAS in South Africa.
